# Autonomic Denervation for the Treatment of Atrial Fibrillation

**Published:** 2011-11-15

**Authors:** Demosthenes G Katritsis

**Affiliations:** Department of Cardiology, Athens Euroclinic, Athens, Greece

**Keywords:** atrial fibrillation, ablation, ganglionated plexi, autonomic nervous system

## Abstract

The influence of the autonomic nervous system (ANS) on triggering and perpetuation of atrial fibrillation (AF) is well established. Ganglionated plexi (GP) ablation achieves autonomic denervation by affecting both the parasympathetic and sympathetic components of the ANS. An anatomic approach for GP ablation at relevant atrial sites appears to be safe, and improves the results of PV isolation in patients with paroxysmal and persistent AF. GP ablation can be accomplished endocardially or epicardially, ie, during the maze procedure or thoracoscopic approaches. Further experience is needed to assess the clinical value of this promising technique.

## Introduction

**Figure 1:**
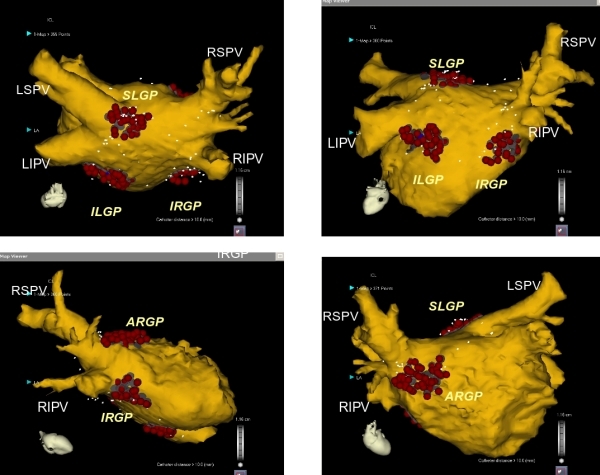
Anatomic position of the major GP targeted for catheter ablation. Presumed GP clusters are ablated 1-2 cm outside the PV-LA junctions at the following sites: *left superolateral area* (superior left GP-SLGP), *right superoanterior area* (anterior right GP-ARGP), *left inferoposterior area* (inferior left GP-ILGP), and *right inferoposterior area* (inferior right GP-IRGP). Another GP (crux GP) the inferoposterior area between the ILGP and IRGP is not indicated. Reprinted from from Katritsis DG et al. Rapid pulmonary vein isolation combined with autonomic ganglia modification: a randomized study. Heart Rhythm. 2011;8:672-8. Copyright (2011), with permission from Elsevier.

The influence of the autonomic nervous system (ANS) on the triggering and perpetuation of atrial fibrillation (AF) is well established. Variations of the autonomic tone have been associated with paroxysms of AF and both sympathetic and parasympathetic activation may be proarrhythmic by shortening of atrial refractoriness.[1-3] Vagal reflexes from clusters of autonomic ganglia, so-called ganglionated plexi (GP), at sites around the circumference of the left atrial-pulmonary vein (PV) junction (Figure 1), may induce and perpetuate AF through spatial heterogeneity of refractoriness.[1,4] In clinical practice, inadverted parasympathetic denervation has been proposed as a potential mechanism of circumferential or antral PV ablation for the treatment of atrial fibrillation.[5,6] Initial studies on partial vagal denervation via epicardial fat pad ablation indicated that such a dedicated approach may prevent AF,[7,8] although results have not been consistent.[9-11] Ablation of areas with prominent sympathetic innervation has also prevented sympathetic AF. The ligament of Marshall is a left atrial epicardial neuromuscular bundle, rich in sympathetic innervation, that has been associated with the genesis of atrial tachyarrhymias and AF.[12-15] Epicardial ablation of the ligament of Marshall in the canine can terminate spontaneous atrial activity and prevent AF,[13] whereas in the human epicardial (through the coronary sinus)[16] or endocardial [14,15] ablation at the insertion site of the Marshall bundle may terminate AF. We know now that selective parasympathetic or sympathetic denervation may not be feasible. Both sympathetic and parasympathetic elements reside in all four major left atrial ganglionated plexi,[16,17] and ablation lesions may unavoidably affect both components of the autonomic nervous system. The area around the ligament of Marshall that has been previously thought to represent an area of predominantly sympathetic innervation has been shown to contain parasympathertic fibers as well.[18] Thus, although initial studies on GP ablation were characterized as attempts at specific parasympathetic denervation, this may not be true. Actually, ablation of atrial areas containing GP unavoidably results in autonomic denervation.

## Techniques of GP ablation

In the electrophysiology laboratory and the operating theater, identification of major GP has been mainly accomplished through high-frequency stimulation (HFS) and induction of vagal responses in the atria.[19,20] HFS is delivered at 1,200 bpm (20 Hz) with a pulse width of 10 ms at 12-24 V.[21] A predominant efferent vagal response is defined as induction of AV block (> 2 sec) and hypotension or prolongation of the R-R interval by >50% during AF, following a 5-second application of high-frequency stimulation. During these studies the anatomic locations of these plexi in the human have been well characterized. However, the method usually entails the discomfort of general anaesthesia, since conscious patients may not tolerate more than 15 V. Furthermore, we have recently shown that that anatomic ablation, ie targeting the areas known to host GP in the left atrium (Figure 1) without previous identification of GP, yields superior clinical results than HFS-identification and ablation of GP in patients with paroxysmal AF.[22]

## Clinical Experience

### Ablation in the Electrophysiology Laboratory

Isolated GP ablation has been employed for both paroxysmal and persistent AF with variable success. In paroxysmal AF, arrhythmia-free survival within the first year after the procedure ranged between 26 to 77%.[22-26] Success rates of < 40% have been reported for persistent AF after a single procedure.[27] GP ablation in combination with pulmonary vein (PV) isolation has yielded better results than PV isolation alone with reported success rates up to 80%.[4,26,28-30] We have conducted the only randomized study that has compared the efficacy of PV isolation vs PV isolation+GP ablation in 67 patients with paroxysmal AF. Recurrence of arrhythmia was documented in 18 (54.5%) patients in the PV group 4.7 ± 1.0 months after ablation, and repeat PV isolation was performed in 7 (21.2%) of these patients 5.1 ± 1.1 months after the first procedure. Recurrence of arrhythmia was documented in 9 (26.5%) patients in the GP+PV group 5.0 ± 1.3 months after ablation, and repeat ablation was performed in 6 (17.6%) of these patients 4.3 ± 0.5 months after the first procedure. At the end of follow-up, 20 (60.6%) patients in the PV group and 29 (85.3%) patients in the GP+PV group remained arrhythmia-free (log rank test, P = 0.019).[29]

### Intraoperative Ablation

Addition of GP ablation to the conventional Maze procedure has produced improved outcomes with success rates 83-93% over the following year.[31-33] Although this approach is interesting it is associated by the risks of open heart surgery and the Maze which nowadays is only considered in patients subjected to valve surgery.

### Thoracoscopic Approaches

Thoracoscopic approaches combine PV isolation with selective GP ablation, with or without ligament of Marshall ablation or left atrial appendage amputation.[34-38] The reported success rates over one year follow-up range from 65 to 86%, and the procedure does not appear to have any associated mortality, with pleural effusion, pneumothorax, hemothorax, and phrenic nerve injury the reported complications so far.

### Complications of GP ablation

Ablation-induced atrial or ventricular proarrhythmia has been reported with both endocardial and epicardial (thoracoscopic) approaches.[28,39-40] Anatomic GP modification appears to carry a higher risk of iatrogenic left atrial tachycardias than PV isolation. In our experience, anatomic or HFS-mediated GP ablation is complicated by atrial macroreentry in 2-10% of patients treated, but does not always require a repeat procedure, since these tachycardias may spontaneously resolve with time.[22,25,27,29]

## The Future

GP ablation appears to be a safe and efficacious method to improve PV isolation in patients with AF, and has been used in the electrophysiology laboratory, and during the maze procedure or thoracoscopic approaches.[41] However experience is limited and mainly based in patients with paroxysmal AF. Furthermore, the long-term efficacy is not known, and this is particularly important since restoration of autonomic activity may occur as early as 4 weeks following ablation.[41] Controlled, randomized studies and more clinical experience are needed to accurately assess the clinical usefulness of this promising technique.
